# Valedictory Address Delivered before the Graduating Class of the Baltimore College of Dental Surgery

**Published:** 1853-04

**Authors:** Philip H. Austen


### ARTICLE III.

*Valedictory Address delivered before the Graduating Class of the Baltimore College of Dental Surgery.* By PHILIP H. AUSTEN, A. M., M. D.

A MAN may redeem the follies of his youth; a science correct the mistakes of its infancy. The arts of medicine and surgery have thrown over the ignorance and misconduct of their youthful days a thick veil of noble deeds, lofty aims, profound learning and heroic self devotion. If to the art of dentistry, youngest in this noble band of three, that stand to minister at the altar of suffering humanity, there still clings some of the reproach of her early associations, it is not because she is more lowly born than they. The deeds of her childhood might well be the boast of riper years; they give sure promise of a maturity of which her elder sisters shall have full reason to be proud. Now, in the broad twilight of the present, to make the darkness of the past forgotten in the brightness of the future, increase onward and upward till it shall reach the fullness of noonday splendor—this, gentlemen, is your mission.

Waste not in eulogy upon your art the precious time that should be spent in acting out its duties: better to show what a dentist can and must be than stop to draw a picture of what he ought to be. The world may admire the picture, but they will judge by the reality. You may prove, by arguments the most conclusive, the necessity of education and science; yet the arguments will weigh little, if the unlettered and ignorant are allowed admission into your ranks. You may talk ever so eloquently about the dignity of your art; yet if, in the pride of your learning, you seek to put away from you one of the duties of that art, as in any wise beneath you—dental science is wounded in the house of her friend, and the blow is the more deeply felt because inflicted by the hand of a traitor.

But gentlemen, graduates of the Baltimore College, you have not so learned the science and art of dentistry. I do only repeat to you some of those truths that we have sought in these halls to impress upon your memory, when I this evening ask your attention to a few thoughts touching the harmony which marks the inseparable elements of your art, its relation to the kindred arts of medicine and surgery, lastly the spirit and the purpose which must attend your life henceforth, if you would seek to honor yourselves and your profession.

John Foster was refused the hand of one he loved, until "he should prove to the world that he was what she knew him to be:" his essays of world wide fame revealed at once the greatness of his mind and the depth of his affection. Gentlemen, your profession asks of you what you should prove yourselves, before the world, to be such as she knows her votaries can and ought to be; until you give this evidence of the strength and reality of your devotion, I sincerely trust she may refuse to you any of the honors and emoluments that are in her gift.

Permit me a few preliminary words upon the title which you purpose to assume. Your diploma styles you "*Doctores Dentium Chirurgiæ:*" terms of complete and significant import. But to their usual translation, Doctors of Dental Surgery; as also to the phrases surgeon dentist, dental surgeon, surgical and mechanical dentist, there are these objections: the three first are inadequate to express the extent of your duties, while the last is either absurd or it implies what is untrue.

Surgery in the full sense of the original "*chirurgia,*" which is precisely rendered by our saxon word "handicraft," covers the entire ground of dental art. Not so, however, the present significance of that word, which the usage of centuries has now so fixed, that it were unwise and unsafe to attempt to alter or extend it. In either sense the skill presupposes knowledge and judgment; but the limitation of that skill, when the word is applied to your art, is fully confessed in the common use of that most awkward of titles "surgeon and mechanical dentist." As properly might you say anatomical and operative surgeon, or pathological and therapeutical physician. We do not say surgical aurist, or occular surgeon: for the aurist and the occulist treat the medical as well as surgical diseases of their respective organs. Therefore to him, who must bring medical, surgical and mechanical skill into such constant and harmonious exercise as your profession requires, that term is most appropriate which will at once express all his duties. This you will find in the comprehensive name "Dentist." Discarding then all phrases of partial significance, let this, gentlemen, be the name which it shall be your delight to honor. Put away from you the insinuation of inferiority which those imply who seek to prefix to it the term surgeon, and acknowledge the right of none to its adoption, who are not qualified in all that it comprehends.

Dentistry, in all its completeness, has so wide a range of affiliations, that should the student confine himself within the limit of its collateral arts and sciences, a lifetime could not complete them. In that knowledge of the entire human system which is essential to the proper treatment of any part he is brother to the physician; in some of his operations, to the surgeon, and in others, to the artisan. The chemist also must be his instructor, and in the cultivation of a correct taste which alone shall enable him to adopt his work to the peculiar expression of his patient, he must learn of the artist and sculptor; not pursuing this, the æsthetic element of his art, in a mere epicurean enjoyment of beauty, but for the higher purposes of usefulness and beneficence.

Among these relatives of dental science, there is one whom, I regret to think, some of her votaries are loth to acknowledge: some who would wish to exclude from the list of essential duties such as are in any wise mechanical. They will have the world to understand that a mechanic and a dental surgeon have very little in common. It is a questionable title of nobility, which will admit of no condescension, and a very feeble character, that cannot impart dignity to what it touches.

But here is no room for condescension; nor may the superiority of such a fastidious delicacy be for a moment acknowledged. If the exercise of mechanical skill is degrading *per se*, then your whole art falls together. There is scarce an operation performed by these surgeons in dentistry, that does not depend for success upon this same despised species of skill. Is it more honorable, think you, to extract a tooth than to replace one: to handle gold foil than fashion gold plate? Yet this is mechanism: that is surgery. Under such a rule the surgeon who by a brilliant operation removes a badly broken limb is more worthy the name than if, by means of some mechanical contrivance, he should aim to save it. God help the patients were such laws of honor rule supreme.

But, see you not, gentlemen, that all our faculties of mind or body are only instruments for the accomplishment of some purpose. If that purpose be lofty, the means used must share the honor; if the purpose be ignoble, so also the instrument. No might of intellect can ennoble pure selfishness, nor any humbleness of means bring a reproach on beneficence. All the appliances you shall bring to the perfection of your art will share in its elevation; whilst, in its incompleteness it can give no honor to its professors.

Why should that mechanical skill be a reproach to you, which is enshrined in the memories of a Watt, an Arkwright, a Fulton and a Whitney: which is inseparable from the fame of Sir Joseph Paxton, Sir Charles Fox or Robert Stephenson? It is no blot on the spotless memory of Newton, that he made a telescope with his own hands; nor does it lessen our estimation of the important discoveries of Lewenhoeck, that his microscopes were the work of his own fingers. Boyle, Davy, Wollaston and Faraday enjoy a higher fame by virtue of that ingenious and skilful dexterity, without the aid of which all the might of their intellects would have failed to unfold many of those hidden mysteries of nature which it is their glory to have revealed. When the brush of the painter, or the chisel of the sculptor shall be thought to tarnish the genius that guides them; then, but only then, may you look down upon the tools, wherewith you must work out the high and useful purposes of your art.

Upon skill apart from its purpose, as upon intellect unapplied, judgment is suspended. In the automaton flute player of

Vaucansen, the wonderful clock of Strasburg and the calculating engine of Babbage we have the same marvelous ingenuity. Yet we view the first with regret, because of its utter uselessness; the second with mixed emotion, for there is here some purpose; but the third with unqualified admiration, for it seeks to achieve great good. Gentlemen, devote your ingenuity to a noble purpose and you need not fear it will degrade you. This false estimate of mechanism, in itself considered, grows out of a natural proneness to give to a thing the rank of its associations. Very properly we judge a man by the company he keeps, and an art by the character of the mass of its followers. Nor should we accuse the world of harsh judgment because the art and the man may perchance be unjust to themselves.

So long as dental practitioners bring to their calling ignorance, with its host of prejudices, and succeed in making the world believe that their malpractice is dentistry—what wonder if men of science refuse fraternity with these, or the world place a low estimate upon such pretension. When the artisan quits a craft of which he is master, and aspires to an art of which he is ignorant, it is not the artisan that is despised, or his skill, but the imposter and his incompetence. The artisan, however humble his position, may be the faithful steward of all the faculties and opportunities given him. Upright integrity may mark his humble course, and as a man, made in the image of his God, it is not for me to look down upon him. Nay, I would clasp the hand of such an one in friendship far sooner than that of many, who, in the pride of intellect, do deeds unworthy of humanity.

*Homo sum et humani nihil a me alienum puto.*

Let not a Christian people be put to shame by a Roman audience, as it rises in homage to this noble sentiment of Terence.

When the mere mechanic lays claim to proficiency in your art without that specific education which its duties require, he at once sacrifices any previously good name by the dishonesty of promising what he cannot perform. It is not his skill that dishonors, but the ignorance that knows not how rightly to apply it. The community at large are not however prepared thus to discriminate. Nor, gentlemen, until you shall unfold the full capabilities of your science, and prove in yourselves what extent of intellectual culture is essential for the mastery of its difficulties, can you be said to have done your part in removing from dentistry the reproach of her infancy. Still more recreant will you be to the trust we this evening impose on you, if, possessed of science and much learning, you imply by your course of action that any thing in the cycle of a dentist's duties is unworthy of your careful attention.

I have called dentistry youngest sister in the great family of the art of healing, and thus expressed the closeness of its relation to medicine and surgery: a relationship which, until recently, they have been loth to own. Whence, may I ask, this reluctance? Not from any disapproval of its end and aims, for they are parallel with their own: not from dislike of the means that must be employed, for they are similar: not because it is a specialty, for their own ranks are full of specialists in highest repute: but because of the want of scientific information prevailing among dentists as a class, the consequent and palpable imperfection of their operations, and because of those acts and modes of practice so common among them, from which a man of true professional pride holds himself carefully aloof as characteristic of charletanism and imposture.

These, the mistakes of unlettered infancy, your profession is rapidly correcting: its capacities are becoming developed with a precocious rapidity no where surpassed in this most progressive of ages and countries. The past ten years have done for dentistry what a century could not accomplish in the younger days of medicine and surgery, and these sciences should remember the follies of their own youth, before they visit with too harsh a judgment the more recent and more venial errors of your own. Surgery was many centuries old, when Fabricius, preceptor of the immortal Harvey, discovered the following *improved* and easy mode of removing a tumor: "If it be movable, I cut it away with a red hot knife, that sears as it cuts; but if it be adhered to the chest, I cut it, without bleeding or pain, with a wooden or horn knife, soaked in aqua fortis; with which, having cut the skin, I dig out the rest with my fingers." When the College of Surgeons of Edinburg was incorporated, it was a prerequisite of membership that the applicant should be able to "read and write, to know the anatomie, nature and complexion of everie member of humanis nature; and lykewayes to know all vaynes of the same, that he may flewbothemie in dew time." If such, gentlemen, were the extent of your surgical knowledge, you would not now be holding the diploma of this College. Medicine too, when far older than your art now is, was rife with abuses greater than any dentistry has ever known; but against them what invaluable discoveries and glorious achievements have we not to offset.

So now is your profession rapidly redeeming the past, and proving her indisputable right to that high seat among the sciences, which she claims in virtue of her noble birth. Men whose talent and education would adorn any calling, she now numbers by hundreds: a few years since they might have been counted by tens. Her work is done with a certainty and success which medicine cannot equal. She is forming a literature of her own; and within her borders are springing up colleges which have already done much, and are destined to do yet more in dispelling the darkness of ignorance, and shedding abroad the light of truth and science.

I speak not these words with the purpose of eulogy upon this, or any other dental college. To their graduates no eulogium is necessary; while to the world, the character of their alumni will more truly tell their worth than any words of praise. I am no friend to that endless multiplication of colleges, each vying with the other who shall make easiest the terms of graduation, and fearing to reject an unworthy candidate least future students shun so strict a school—until a diploma is scarce worth more than so much parchment, the name Doctor no warrant of much learning, and the title of Professor no guarantee of any peculiar talent. Of medical schools our favored land has quite a sufficiency: but the right training of the dental student is in so great a degree distinct from that of the tyro in medicine, as to make imperative the demand for separate colleges of dentistry. Perhaps I cannot better express my views of the true relation between the three branches of the healing art than by a brief consideration of this necessity.

The peculiarities of dentistry which call for this separateness of instruction are first its specialty, secondly the great importance of manual dexterity: the latter demanding a new adjustment of time; the former a new direction of general studies. Take any of the several branches of medical science and the full mastery of that one demands that it shall be the prominent subject of study during a lifetime; especially if you seek to penetrate beyond what is known into the region of discovery. True, the need for this concentration of thought is a confession of the imperfection of our faculties; but in its very universality it ceases to be humiliating. Few indeed are those minds of giant grasp of whom it may be said *"nihil erat quod non tetigit, nihil tetigit quod non ornavit."*

Therefore in either the elementary studies, anatomy, physiology, botany and chemistry; or their more complex and more practical modifications, surgery, therapeutics, pathology and materia medica, thorough instruction is altogether impossible, whether we make the term of study three years, seven years, or a lifetime. Partial teaching is all that the most admirable school can give, and this best, when the general outlines of each science are filled in with a minuteness, proportioned in its nature to the profession to be taught, in its degree to the time spent in preparation. In this harmonious adaptation of studies to the end sought, much judgment is called for. If the subjects taught are too few, knowledge is defective: if too many, it becomes superficial: and if not adapted to the profession chosen, it is unpractical. Greatly is the difficulty increased if the term of study is shortened or if several pursuits are attempted to be taught in one.

Five years would not be misspent in the prosecution of those studies which go to qualify the physician or the surgeon for the practice of their respective duties. But the sphere of each, however much they may have in common are yet widely different, requiring difference of talent, and in these five years of preparation demanding difference in the books to be read, lectures to be heard and clinical practice to be witnessed. In England and Europe the distinctness of these professions is maintained in their education, their titles, their societies and their sphere of practical duties. But in our country, from the operation of causes into which it is not now the fit time nor place to inquire, this separateness is maintained neither in education or in practice. That this is not right needs perhaps no better proof than the existence of one or more physicians in every city or community, to whom the acknowledgment of peculiar fitness has assigned a practice especially surgical. Who does not know that our first surgeons are seldom our best physicians? No science can boast many Macaulays or Broughams, the healing art has few of such "double first class men." To these who, by intuitive address, open the treasuries of art and skill, it is permitted to range over the whole cyclopedia of knowledge: but upon those, who only by dint of patience and perseverance can force back the stubborn lock of learning, it lies incumbent to give a resolute concentrated purpose to the chosen duty of life, whether in the study that is to fit for action, or in the action itself. If then so apart lie the work of the surgeon and the work of the physician, that talent for one does not insure success in the other; so wide their several spheres that the lifetime and genius of no one man can raise him to highest excellence in both; so diverse in much the training for either, that a due economy of time demands an education for the former separate from the latter: do I claim aught that is unreasonable or absurd for dental science, embracing as she does a third class of characteristic and essential duties, when I ask that she shall be allowed, in colleges of her own, to adapt the elements of instruction to wants of which she is the best judge, and so as to promote an honor of which she is the proper guardian?

If again, in a five years course of tuition, this modification of the principles of the healing art, according to the wants of its several practical departments, becomes necessary, in view both of a wise economy of time and fitness of preparation; *a fortiori*, is it essential when the impatient haste of Americanism reduces that course to four, three or two years, and makes the last the most common period of study. True such haste may not be defended. The cautious Englishman may err in giving those years of his manhood to the study of the experience of others, which might better be spent in treasuring up the more impressive and better remembered lessons of his own. But when the American, eager for action, goes forth to the contest with pain and disease, before he has learned so much as the general principles of this art of war, too often the graves of his friends will be thicker on the field of battle than those of his foes. The charge of life and health is not so light that it may be assumed without preparation, or so easy that it may be learned in a day. Our prominent medical schools, in whose power it is to control the period of preparation, incur a fearful responsibility, when, in the spirit of unlawful rivalry and for the sake of unholy gain, they send forth men who, they well know, have not had time to become what their diplomas proclaim them to the world to be.

Gentlemen, wide is the field of your usefulness, many and difficult your duties, to the mastery of which you must bring talent and industry. But you are seldom called to sickness which is unto death; the issues of life do not often hang on your operations; you are spared the painful responsibility of knowing that upon the casting vote of your skill depends under God the even tie of existence. Therefore we cannot ask of the dental student a four or five years noviciate, when that of the medical student extends over but two or three. We can only demand as long a stay in our halls as they ask in theirs. We make you as competent to your art, as the newly fledged physician to his. Neither can do more than lay the groundwork, and give the plan and specifications for the superstructure. Let the student in each seek to build upon that basis which has been laid for him; otherwise he shall find here and there parts of his edifice crumbling for want of support. Believe me, gentlemen, the best planned foundation, on which for you to build a fair proportioned excellence, is to be found in a well ordered, well adjusted, well sustained dental college. It does not here become me to say how far the Baltimore College fulfils these conditions.

The inadequacy of medical schools to give even the scientific proficiency you require, is evinced in the ignorance their graduates show of even the purposes of your art, much less its practice. The few simple operations which the country physician is called upon to perform, are done with a want of tact and judgment, which leads us to hail with pleasure a recent movement made to establish a dental lectureship in the principal schools, whereby some rays of light may shine in upon this Ægyptian darkness. The Baltimore Dental College will cheerfully hail such an appointment in the University of Maryland, nor deem that in so doing she is encroaching upon her own province of instruction. But it is not by any one such chair, that medical schools can alter that constant application of the truths of science to the special wants of their students, which marks their whole course of teaching: which in fact gives to that teaching its greatest value. Some of these specialties of medicine are of secondary value to the dental student only because less essential than others, and in the limited period of his schooling they must therefore give way to what is more necessary to be learned to fit him for practice. In the prosecution of your studies hereafter, you will so seek to enlarge the boundaries of your knowledge, as to take in, not these alone, but many other themes which we can, in the short months here allowed, only point out to you as worthy your careful study. When, in favor of this pursuit of details which one will never be called upon to apply, it is urged that, if he does not do so when a student, he never will when a practitioner, there is that implied, of which I trust, gentlemen, you will prove the injustice and untruth. If you suffer yourselves to become so engrossed by your professional duties that you have no time or inclination to carry a subject, so closely allied as medicine, beyond the limit of your own practical use thereof, I have no idea you will do so with any other more remote science; nor can you ever stamp upon your profession that mark, which the man of cultivated mind and enlarged acquirements is enabled to give it. All the cramming of a dozen medical courses would fail to redeem you from such a position, whether placed there by indolence, incapacity, or love of mere pecuniary reward. Such knowledge as is requisite for the commencement of a correct practice, we have sought to teach, in due measure and harmonious proportion; while much that will give increased beauty and dignity to that practice, we have only pointed out for your future study. No system of instruction will suffice for your training, if there dwell not within you the power and the will to search out the deep things of science beyond their mere evident application to the necessities of your art. We cannot create a soul of genius under the lifeless ribs of dullness; awaken the kindling spark of ambition beneath the dead ashes of sloth; or impart the warmth of benevolence to the cold heart of selfishness.

Against private instruction I have nothing to urge. In either profession it is an undeniably valuable assistant to that system of collegiate tuition which each demands. But the generalities of a medical education cannot be made available to the dentist by a few months private office instruction; nor is the dextrous ingenuity of your art so readily acquired, that it may in any justice be set aside as an after thought of easy mastery. A medical diploma might give warrant of your capacity as a physician, but of itself should be accepted as no token of your fitness for the specific duties of dentistry.

While your profession had no schools of its own, its members might be excused for protecting themselves from the charge of ignorance by wearing the badge of a sister art; and American surgery with no separate schools must still do the same. But does the absence of an M. D. from the names of Liston, Brodie, Cooper and Hunter, imply any defect in the education of those bright stars of English surgery? It is time the American people should begin to understand that there may be a title more expressive of your proper qualification than the one of Doctor in Medicine. They have defined your duties for you, as distinctly as the English have defined the surgeon's duties for him: they must also demand a like separateness of preparation. Such a course of study we have sought to mark out. By virtue of this we deem you prepared, in both the science and art of dentistry, to commence its practice, and the peculiarity of your fitness is expressed in your peculiarity of title. If, gentlemen graduates, you seek to add to this title another which you imagine more honorable, instead of proving to the world that yours is the best guarantee of proficiency, you become recreant to the cause of your alma mater; false to your noble profession, whom you thereby confess to be incapable of teaching her own practice and principles.

Let us turn now for a few moments from the science to her votaries: from the art to the artist: from dentistry in the abstract to that concrete reality, which each of you severally shall make it, for it is upon this reality that the world will base its judgment of the honor, usefulness and rank of your profession. Remember that dental science is young and has yet to achieve distinction for herself. Her nobility of soul will never suffer her to owe position to her sisters' greatness, or sit content in the reflected light of their brilliant achievements. She acknowledges no splendor of name to gild profligacy or crime; no inheritance of past deeds to be squandered in idleness; no historic renown to cover ignorance and stupidity. Stripped of the false glare, which some titles shed on worthlessness, you have so far the advantage of the physician, that you have no temptation to rest in a name that you have not earned, or boast a dignity which you do not deserve. Another advantage also you have, in that the success of your labor is more sure, and its appeal to the community more direct: they can more accurately judge of your merit, and your reputation is not so much the thing of chance and caprice. Want of success may be a physician's misfortune, but the sin of your failure will lie at your own door.

He who would hope to enjoy these advantages must be an earnest, willing worker. Labor is the lot of man: to some a blessing and to some a curse. The labor of mere drudgery and the harder toil of indolence is a grievous burden: but for him who, tasking his energies for some high and holy purpose, goes forth to a life of usefulness, it is sweetened by duty and rewarded by honor.

"That, like an emmet, thou must ever toil
Is a sad sentence of an ancient date,
And certes there is for it reason great:
For though it sometimes make thee weep and wail,
And curse thy stars, and early rise and late,
Withouten that would come an heavier bale,
Loose life, unruly passions, and diseases pale."

In your ranks there is no room for the sluggard, no place for the mere dilettante. The vain and empty trifler, indulging in idle sentiment, or in wild speculation, with no higher end before him than self indulgence, and failing to accomplish any work that may promote the welfare of his race—the humblest laborer that goes forth in cheerfulness to his daily toil, is a greater, because more useful man than he. There is a task of difficulty before you, and with a brave strong heart you must advance to its mastery. If ye are men, with the energies and affections of men, healthful, strong, true hearted men, no difficulties shall stay your onward march; nor shall the fair domains of your science much longer lie under the scourge of the rampant Minotaur of ignorance.

In this devotion to your cause, I do not ask you to neglect self interest, or repress aspirations after fame. But true self love has no sympathy with blind selfishness; a lofty ambition has nothing in common with a vulgar thirst for notoriety. He who is greedy of applause is in danger of seeking favor by unworthy means; nor, while drinking in deep draughts of praise, will be apt to take heed how the purity of the waters is tainted by the source whence they flow. He again whose thoughts rise no higher than his own interests; who views his art only as a mint for the coinage of gold, and values his patients by the amount of their contributions to his treasury—such a one is no object of envy, had he the wealth of Crœsus; no honor to your science, though he live in all the splendid magnificence of Lucullus.

Money is a legitimate object of your pursuit: but it will flow in upon you in sufficient measure without your making it the only or even the prominent thought. Seek the utmost perfection of which your art is capable, and let every case you touch come up to that high standard. Then place upon your work a remunerative value. Your charges may be threefold greater than your neighboring rival—what matter that, if your work be tenfold more lasting? Your profession is not a trade of petty bargains. It is knowledge and skill which you sell; health, permanent cure and comfort which your patients buy. You alone know the worth of what you give; time only can show them the full worth of what they receive: there is here no ground work for bargaining. Let your services be of highest excellence; and see that you suffer neither yourself nor others to undervalue them. Rest assured that by such a course of uncompromising independence, if tempered with courtesy and sustained by merit, your practice will, in the end, be the larger, yourself more esteemed and your profession more honored.

If on the other hand you seek to gain practice by underbidding your rivals, rather than by the intrinsic worth of your operations; if you undertake more cases than your time will allow you properly to perform; if you neglect the more difficult and delicate points of your duty, for fear of insufficient compensation; or so engross your hours with the practice of your art, as to leave you no time to inform yourself in the study of its science—no words of praise from your lips can wipe out the stain of your actions. To learn your duty in its fullest detail will fail to ennoble you, if from its faithful performance you turn in any wise astray, led by the love of gain, or the promptings of a false self interest. The errors of ignorance are more excusable than these.

Hold no secrets in your art. Freely you have received, freely give. A glance at the past history of your profession will show you what a withering blight this narrow minded, short sighted policy has thrown upon its growth, and ought, if you have any love for your art, to make you forever eschew it. If dentistry is no more than a money making craft; then patent rights and secret inventions are not out of place; but if you claim for it the rank of a science, you must act out the generous liberality of men of science, and each bring cheerfully his own deposit to the great storehouse of knowledge. Be assured that you shall receive tenfold for all you give. Send forth the treasured secret; the greater its value, the greater the beneficence, and you will be spared the just reproach of suffering humanity, in whose cause you profess to have enlisted. "There is that scattereth and yet increaseth: there is that withholdeth more than is meet, but it tendeth to poverty."

To diligence, faithfulness and generosity, forget not to add that spirit of courtesy, which refines the lowliest, and clothes the highest intellect with a most delicate grace and fascination. Not the polite condescension, which insults its object; nor the servile flattery, which degrades the one who offers it: not the weak acquiescence of timidity, which shrinks from opposing error; nor yet the hollow form of words and manner, covering indifference or enmity—not these base counterfeits would I here commend to you; but that most genial courtesy of kindliest influence, that springs from a heart full of love for the good, the beautiful, the true; in which is wide room for the sympathies of a broad spread humanity.

In your intercourse with your professional brethren, in that respect which is due to their differences of opinion, you will meet abundant opportunity for its exercise. Oneness of aim does not imply identity of means; nor would a tame monotony of thought and action be for one moment desirable. There is a lethargy more deadly than strife, and a discussion whose kindly flow forbids all anger, if only you will extend to the sentiments and feelings of others that candor and courteous forbearance which you would demand for your own. To expose a brother's falsehood is not the best proof of your own truth; nor to display his weakness, the highest evidence of your own strength.

But, gentlemen, I would ask you to extend this courtesy beyond the pale of your profession. When you come in contact with the uneducated empiric, it is neither kind nor wise to meet him in an angry, a defiant, or a contemptuous spirit. Not kind, for his position may be his error not his vice: not wise, for, without serving your profession in the least, you may make a life long adversary for yourself. Strive to gain friends; foes will spring up without being sought out.

If truth be assailed, defend it with a strong heart manfully: but see well to it that you do not confound a just anger against error with an unholy hatred of the offender. Remember that he who needlessly makes truth disagreeable, commits high treason against virtue: nor can you do a much more grievous wrong to the cause of your science than to stain her defence with the bitterness of personal invective. It will in no wise excuse you, that you fight only with the weapons of your opponents. To all such attacks silence is the best reply; an upright life their completest refutation. In their secret hearts all men most admire the calm self sustained courage that can walk unanswering amid calumnies.

It may be true that the community, among whom you seek to cast your lot, are the victims of grossest charlatanry; but rudely to tell them this is no sure or pleasant way to do them good. Go forth strong in the love of your art, and in its faithful practice you shall do more for yourselves and others than by volumes of abuse. Entertain for it that high admiration, which it shall be your just pride to win for it from others. Pursue with diligence the many branches of science which are essential to the full mastery of its difficulties, and discharge its duties with an unswerving integrity which looks beyond the mere question of present reward.

But above all seek to engraft upon this your high professional character, the still nobler elements of a high moral character. Stamp deep the inner and the outer life with the unfading mark of that signet whose glorious impress is the "Beauty of Holiness." Win for yourselves that name than which there is none more honorable or of higher significance—the name of "Christian gentleman." So blending official, social and religious duty—your profession shall acknowledge you with pride; the community in which you dwell shall honor you; and that true fame, which lies in the just appreciation of your worth, shall come to give you added happiness and encouragement. Upon a youth of temptation resisted, and a vigorous manhood of duty fulfilled, will follow a serene old age of pleasant memories, cheered by an undying hope, whose lustre shall brighten when the glories of earth grow dim in the waning light of life's setting sun.

And now,

GENTLEMEN OF THE GRADUATING CLASS—We have but to speak the parting word, the farewell that must be, and yet lingers. May these months of a now severed relation come to you in after years, as fruitful in pleasant recollections, as your respectful attention and unvarying courtesy have made them for us. May your life redeem its promise of usefulness, and your success fulfil the hope, which your past zeal and diligence have justly awakened. Not in sad thought at the loosing of pleasant ties would we utter our farewell: but with words of good cheer and encouragement we usher you upon the broad arena of professional life, and bid you a hearty God-speed.

